# Long-term *ex ovo* culture of *Caenorhabditis elegans* embryos

**DOI:** 10.1101/2025.06.26.661779

**Published:** 2025-08-28

**Authors:** Clover Ann Stubbert, Cherry Soe, Pavak Kirit Shah

**Affiliations:** 1. Molecular Biology Institute. University of California, Los Angeles. University of California, Los Angeles. Los Angeles, CA 90095 USA; 2. Department of Molecular, Cell and Developmental Biology. University of California, Los Angeles. Los Angeles, CA 90095 USA; 3. Institute for Quantitative and Computational Biosciences. University of California, Los Angeles. Los Angeles, CA 90095 USA

## Abstract

The genetic tractability, transparency and invariant development of the *C. elegans* embryo have led to its broad adoption as a model system for the study of cell and developmental biology. Its impermeable eggshell has restricted the use of small-molecule interventions during embryogenesis. Existing genetic approaches for rendering the embryo permeable to acute small molecule treatment have increased the accessibility of early embryogenesis of pharmacological manipulation but compromise long-term viability for use in studies of later developmental processes or post-exposure physiology. Here, we describe the use of an optimized enzymatic eggshell digestion protocol coupled with a minimal, serum-free culture media that supports the survival and normal development of *ex ovo* embryos through larval maturation and adulthood. We show that this approach renders embryos permeable to a wide range of small molecules, enabling precise temporal manipulation of developmental processes previously inaccessible through conventional genetic or physical methods. We demonstrate the utility of this technique through the application of small molecule fluorescent dyes, the pharmacological modulation of key cytoskeletal components including microtubules and actin, as well as the minus-end-directed microtubule motor protein dynein, highlighting applications for study of cell division, morphogenesis, and neuronal cell biology, especially in later stages of embryogenesis. This approach expands the experimental toolkit available for labeling and manipulating developmental processes in *C. elegans*.

## Introduction

The nematode *Caenorhabditis elegans* is a powerful model system for the study of genetics, development, and cell biology owing to its genetic tractability, transparency across life stages, and invariant development. In contrast to the amenability of the *C. elegans* embryo to live imaging^1,2^, advances in the perturbation of developmental processes have been primarily limited to genetic^3,4^ and optical approaches^5–8^ by the impermeability of its eggshell, consisting of multiple proteoglycan and chitin layers^9^. This renders *C. elegans* embryos impermeable to most small molecules, perhaps most famously allowing them to survive exposure to germicidal bleach^10^.

A genetic screen previously identified multiple genes implicated in the eggshell permeability barrier^11^. Knock-down of these genes by RNAi causes the generation of embryos whose eggshells are permeable to some dyes and small molecules, which has enabled useful pharmacological manipulations of the early embryo^12–16^. This approach has some drawbacks, as it results in small brood sizes and compromised viability with embryos typically failing to develop past the first two cell divisions^11^. Physical methods of permeabilizing the eggshell (laser ablation^17,18^, coverslip pressure^19^, and microinjection^20^) also have been used to introduce small molecules into *C. elegans* embryos but are limited in throughput, reduce viability, and exhibit variability.

Cell culture systems are inherently more compatible with small molecule treatments and the *in vitro* culture of *C. elegans* embryonic cells has been previously demonstrated. Generally, these approaches involve the recovery of embryos by dissociating gravid adults using alkaline hypochlorite followed by eggshell removal via digestion by chitinase. The exposed embryos are then released from the peri-embryonic layer and disaggregated by shear stress (such as by aspiration through a fine needle) to then be cultured in a range of media conditions that can support the development of early blastomeres^21^ or the growth and survival of differentiated neurons and muscle cells^22^.

We reasoned that an approach based on these cell culture media may be able to support the long-term culture of *ex ovo* yet intact embryos in the absence of an eggshell. Indeed we have found that optimized eggshell digestion protocols, when combined with a simplified serum-free culture media, supports the survival and growth of embryos through larval maturation, producing healthy individuals that are able to be transferred to standard nematode growth plates and raised to fertile adulthood. We show that these cultured embryos are compatible with manipulation by many common small molecules (eg. dyes, toxins, and selective inhibitors) at developmental stages challenging to access by previous approaches. We demonstrate that these treatments can be used to manipulate a wide range of biological processes and structures within the *C. elegans* embryo including the microtubule and actin cytoskeleton and the activity of the minus end-directed microtubule motor protein dynein. This work provides a scalable approach that opens new possibilities for the labeling and manipulation of the *C. elegans* embryo for studies of development and cell biology.

## Results

### *Ex ovo* culture of *C. elegans* embryos

Genetic perturbations that render the eggshell permeable to small molecules also reduce viability^11^, making these approaches incompatible with studies of developmental processes that occur later in development such as tissue morphogenesis and neuronal development. We hypothesized that media optimized for nematode embryonic cell culture may support embryo viability in the absence of the eggshell permeability barrier. We use a simplified version of a culture media originally developed for maintaining *C. elegans* neuronal and muscle cells in culture^22^, omitting the serum the original protocol used so as not to provide the intact embryo with exogenous growth factors. This minimal media is prepared from commercially available Leibovitz’ L-15 media supplemented with 7.7 g sucrose / 500 mL and 1× penicillin and streptomycin (this can be omitted for short-term culture).

To test the ability of this media to support embryo viability, we first prepared embryos for digestion by collecting worms and embryos from a 60 mm nematode growth media (NGM) plate and removing excess *E. coli* by washing with M9 buffer after pelleting worms and embryos by centrifuging for 7 s in a swinging bucket centrifuge (reaching a max speed of approximately 2000 RCF). The pellet was then treated with alkaline bleach wash to digest intact adults, freeing their eggs. The collected embryos are pelleted by centrifugation again for 7 s, and the pellet was washed 3 times with the supplemented L-15 media. We optimized eggshell digestion using yatalase^23^ (T017, Takara Bio), but have found that more inexpensive sources of chitinase, such as from *S. griseus* (C6137, Sigma-Aldrich) also work well. Stock solutions of enzyme were prepared at 10 mg/mL in supplemented L-15 media and frozen in 10 **μ**L aliquots. After thawing and warming to room temperature, 50 **μ**L of the washed embryo suspension is added to the media and embryos are left to rock for up to 1 hour. Digested embryos were transferred by capillary pipette into a 100 **μ**L droplet of fresh media and covered in mineral oil to prevent evaporation and left overnight at room temperature (21–22 °C).

**Figure 1.**
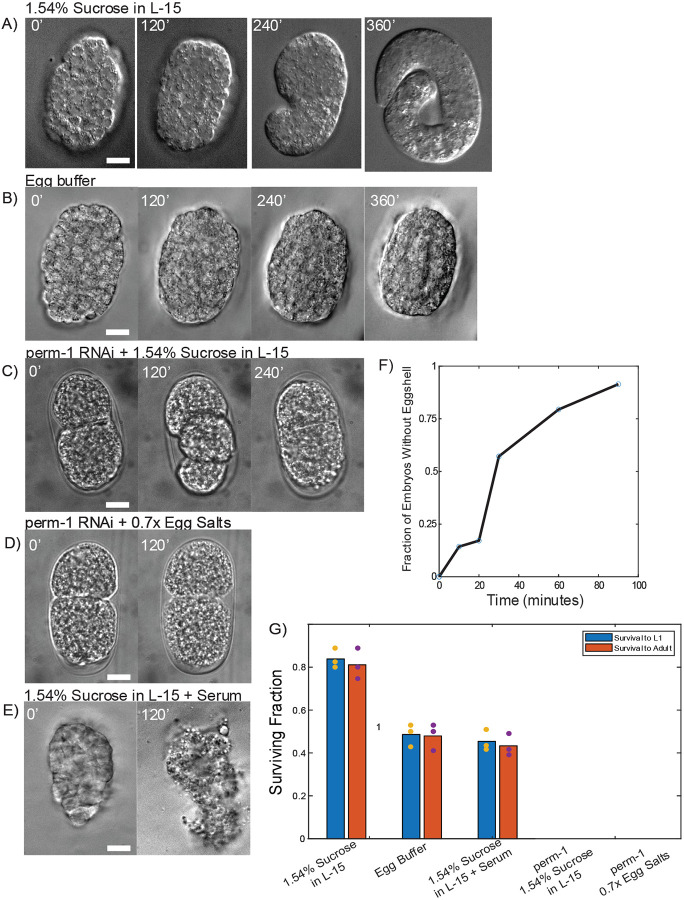
A) *ex ovo* embryos develop normally in 1.54% sucrose in L-15 (10 μm scale bar). B) *ex ovo* embryos show signs of osmotic stress when left in standard egg buffer and arrest (10 μm scale bar). C) *perm-1* RNAi embryos complete 1–2 rounds of division before arresting in supplemented L15 media. D) perm-1 RNAi embryos arrest after 1–2 cell divisions in 0.7x egg salts. E) *ex ovo* embryos in 1.54% sucrose L-15 media with serum quickly arrest and undergo apoptosis (10 μm scale bar). F) Time course of eggshell digestion in yatalase. Error bars show standard deviation of three replicates (with 18, 19 and 22 embryos in total each. G) Survival rates of *ex ovo* embryos cultured overnight in 1.54% sucrose in L-15, egg buffer, and serum supplemented L-15 media and perm-1 RNAi embryos cultured overnight in supplemented L15 and 0.7x egg salts. Blue bars show survival to L1 while orange bars show survival to fertile adulthood.

After digestion, the eggshell is clearly missing when observed under high magnification DIC microscopy, but the embryos remain encased in a soft membrane we attribute to be the peri-embryonic layer observed in electron micrographs of the eggshell^9^. After digestion, comma stage embryos will extend their tail less tightly folded and 3-fold embryos appear spherical rather than ovoid. Upon completing embryonic development, L1 larvae are able to hatch from this soft membrane as normal (Fig 1A). We observe that >80% of early stage embryos (younger than bean stage and thus lacking an epidermis at the time of eggshell digestion) are able to survive and hatch as L1 (40/50) (Fig 1F). Many of the embryos that fail to hatch show signs of mechanical trauma, likely suffered during the transfer process. We recovered a portion of the L1 larvae onto standard NGM plates seeded with OP50 *E. coli* and observed that >90% of those survived to become fertile adults (28/30) (Fig 1F). Late stage embryos are approximately as successful in reaching L1 (35/50) and becoming gravid adults (29/31), demonstrating that this minimal culture media is capable of maintaining embryo viability after eggshell removal and that animals treated in this fashion are fully capable of surviving and maturing to adulthood. The few larvae that are lost were not found arrested or dead, so were likely lost after crawling up the petri dish wall or under the NGM layer. Unsupplemented L-15 and standard egg buffer^24^ yield a much lower rate of survival with many embryos showing signs of osmotic stress (Fig 1B–C).

Given the poor survivability of *ex ovo* embryos in egg buffer, we next asked whether the early arrest of *perm-1* embryos in prior work might be rescued by supplemented L-15 media. While all embryos (25/25) eventually arrested, the embryos typically completed more cell divisions than in egg buffer, suggesting that *perm-1* RNAi itself has significant negative effects on embryonic viability (Fig 1D–E).

We next optimized eggshell digestion duration and yield. By observing embryos in the yatalase solution periodically under DIC microscopy, we tracked the progression of eggshell digestion over time. Just over half of the embryos are digested within 30 minutes with an increase to roughly 90% of embryos digested within 90 minutes (Fig 1D). For experiments where the experimenter can manually sort fully digested embryos from those with remaining eggshells, shorter digestions are sufficient. More complete digestion can also be achieved by preventing the saturation of the enzyme suspension by digested chitin. We accomplished this by replacing 20% of the yatalase suspension with fresh enzyme solution after 30 minutes of incubation. This strategy reduces the survival rate of embryos to 58% for early stage embryos (29/50) and 54% for late stage embryos (27/50) but of those that survived to L1 roughly 90% of both early and late stage embryos became gravid adults (E=27/29, L=23/27). We attribute the drop in survival rates to the extra handling of the embryos and additional shear stresses during the exchange, as even partial eggshell digestion renders embryos extremely fragile and the loss of adults to larva crawling up plate walls as no arrested or dead larvae or adults were ever observed on plates.

### *Ex ovo* embryos are permeable to fluorescent dyes

Advances in synthetic chemistry continue to produce a wide range of small molecules that exhibit both desirable photophysical properties and selectivity to diverse subcellular compartments or sensitivity to variation in local environment. The loading of cell permeable dyes into the embryo has previously been achieved under genetic^9^ or coverslip pressure^19^ permeabilization of the eggshell, or by feeding/injecting adults^25,26^. We reasoned that enzymatically digesting the eggshell would similarly render the embryo accessible to fluorescent dyes but in a manner compatible with long-term viability when combined with supplemented L-15 media.

**Figure 2.**
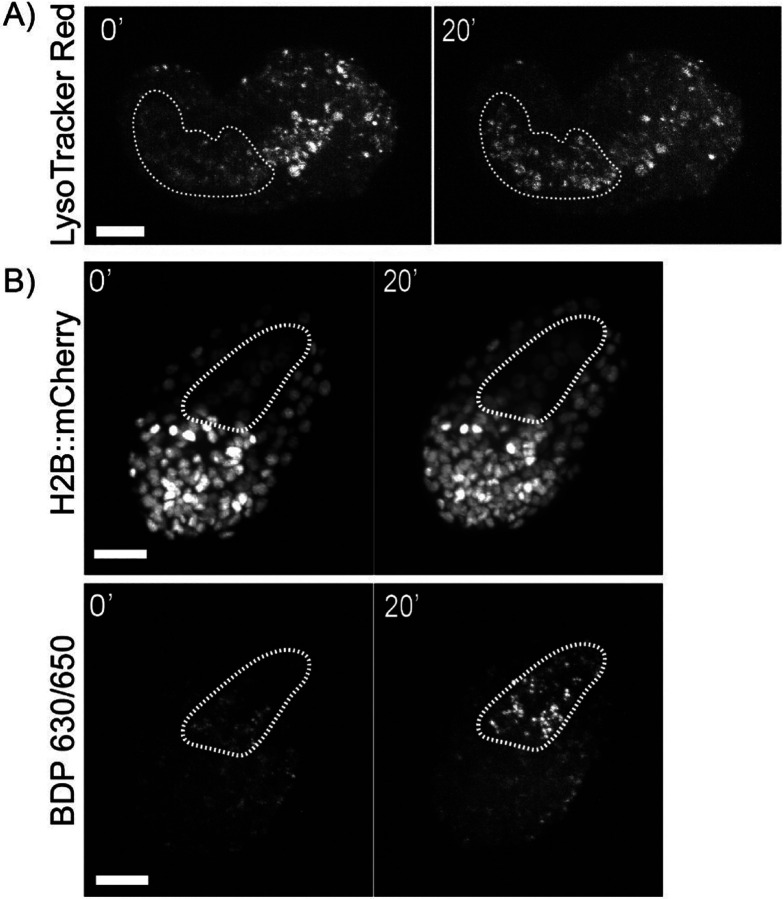
A) Maximum projection of *ex ovo* embryo stained with LysoTracker Red (10 μm scale bar). B) Maximum projection of *ex ovo* embryo stained with BDP 630/650 (10 μm scale bar). Dashed line outlines the embryonic intestine.

Lysotracker^27^ is a hydrophobic weak base that accumulates predominantly in acidic endosomes and lysosomes. This dye has been previously used in *C.elegans* studies to study gut granules and neuronal signaling in embryos and the adult worm by feeding worms on NGM plates with LysoTracker Red added for 12–48 h.^28^ In *ex ovo* embryos cultured in L-15 media, Lysotracker rapidly stains all embryonic cells at all embryonic stages. Labeled lysosomes can be seen throughout the tissue with an increase in the number of lysosomes observed in the embryonic pharynx and intestine (Fig 2A). At 100 nM, the manufacturer’s suggested concentration for staining mammalian cells, LysoTracker Red exhibits toxicity as all stained embryos arrested after roughly 1 hour. At 10 nM, embryos developed normally and survived through elongation and twitching while acquiring sufficient fluorescence to capture with confocal microscopy.

We then replicated prior work staining neutral lipids in *C. elegans* embryos with a Boron-dipyrromethene (BODIPY) dye, using BDP^™^ 630/650 (Lumiprobe Corporation) to stain *ex ovo* embryos. Boron-dipyrromethenes are a class of lipophilic fluorescent dyes used to stain cell membranes and lipid droplets^29^. Similar to LysoTracker Red, we see that BDP^™^ 630/650 rapidly labels all cells in *ex ovo* embryos. As with prior work with chitinase-treated embryos^30^, we observed both weak plasma membrane staining as well as enrichment of the dye in droplet-like structures. At the late comma stage coinciding with the terminal divisions of the endodermal E lineage, we observed a rapid enrichment of both LysoTracker Red and BDP^™^ 630/650 staining in the embryonic intestine (Fig 2B).

### Manipulation of microtubule stability in *ex ovo* embryos

The microtubule cytoskeleton plays a critical structural and functional role in a wide range of fundamental cellular processes from mitosis^31^ to morphogenesis^32^. Genetic and biochemical studies of *C. elegans* have identified a range of functional regulators of microtubules^33,34^. The essential role of microtubule regulation creates challenges for the study of the cytoskeleton at later developmental stages. The incomplete permeability barrier of the early single cell embryo and laser microbeam based permeabilization have previously been used to deliver microtubule toxins and drugs targeting microtubule regulators^11,35^. Given the accessibility of our cultured embryos to dyes, we reasoned that this approach would also make it possible to utilize microtubule toxins to manipulate the cytoskeleton at a wider range of developmental stages.

**Figure 3.**
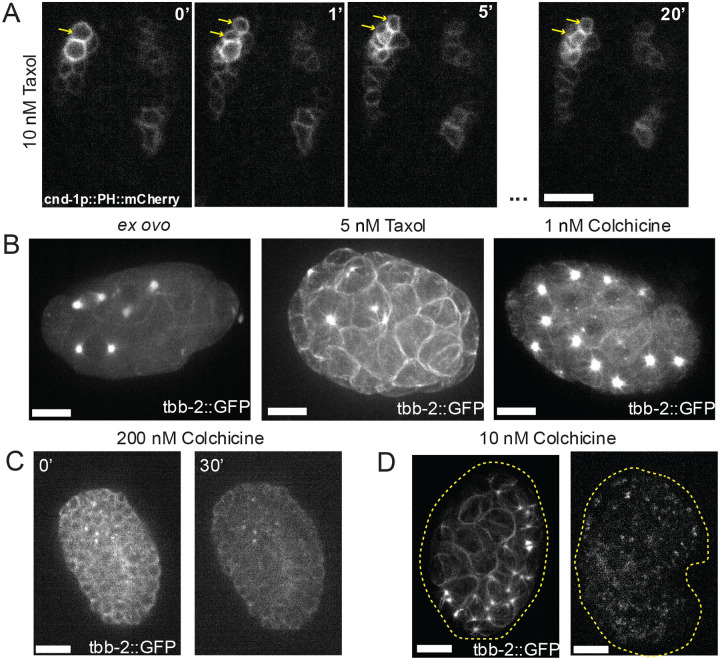
A) Maximum projections of cnd-1p::PH::mCherry embryos treated with 10 nM at the time of treatment, one minute post treatment, two minutes post treatment and twenty minutes post treatment. Yellow arrow heads pointing to cell division post treatment. B) Maximum projection of tbb-2::GFP early stage *ex ovo* embryo in the absence of toxin (left). Maximum project of tbb-2::GFP early stage embryo treated with 5 nM Taxol (middle). Maximum projection of tbb-2::GFP early stage embryo treated with 1 nM of colchicine (right). Image intensity scaling is identical for all three images. C) Maximum projection of an tbb2::GFP embryo in the presence of 200 nM colchicine at the time of drug addition and 30 minutes post drug addition. Image intensity scaling is identical for both images. D) Maximum projection of early (left) and late (right) stage embryos tagged with tbb-2::GFP treated with 10 nM colchicine. Dashed line outlines the embryo. Image intensity scaling is identical for both images.

Taxol functions by stabilizing microtubule filaments, preventing them from depolymerizing and stopping cell divisions causing the cells to apoptosis^36^. This treatment has previously been used to study how microtubules affect the axis of the first cell division of the embryo and used as a control when testing new destabilizing treatments^35,37^. We tested a range of toxin doses in *ex ovo* embryos. Starting with a dose that had previously been used with *perm-1* RNAi treated embryos (100 nM) caused the cells in embryos at all developmental stages to immediately take on a spherical shape and arrest and the same effect being seen at 50 nM. The addition of 100 nM taxol to embryos with intact eggshells caused no visible phenotype leading us to believe it is not able to penetrate the eggshell. Lower doses (10 nM) slowed the onset of complete stabilization treatment and allowed for the execution of a single post-treatment round of cell division to be completed before arresting completely, raising the possibility of studying the consequences of intermediate levels of microtubule stabilization (Fig 3A). Using even lower doses (5 nM), we observed a large increase in the amount of tubulin associated with the mitotic spindles in early stage embryos, with them pulled outward to the cell cortex (Fig 3B). At the lowest dose we tested (1 nM), cell divisions progressively slowed over the course of an hour until, regardless of embryo stage, all embryonic cell divisions stopped.

Colchicine is a microtubule depolymerizer at high doses and a stabilizer at low doses^38^. In adult worms, colchicine has been used to disrupt dendritic microtubules in sensory neurons to determine their function in sensitivity to stimuli^39^. At 200 nM, a concentration used previously in *perm-1* RNAi treated embryos^11^, embryonic cells rapidly became spherical and eventually burst (Fig 3C). β-tubulin fluorescence in these embryos became extremely diffuse and cytoplasmic compared to the cortical localization observed in untreated embryos. This dose causes no visible phenotype in eggshell intact embryos. At a lower dose (10 nM) later stage embryos after hypodermal enclosure continued development for a short period of time (roughly 30 mins) with β-tubulin becoming less localized at the cell cortex over time before arresting, while younger embryos showed the opposite phenotype with β-tubulin increasing at the cell cortex and the embryos arresting immediately after treatment (Fig 3D). At 1 nM, Colchicine mimicked the effects of Taxol treatment and appeared to stabilize microtubules, causing a progressive accumulation of β-tubulin at mitotic spindles and a slowing of cell divisions leading to complete arrest within 30 minutes (Fig 3B).

### Manipulation of actin in *ex ovo* embryos

**Figure 4.**
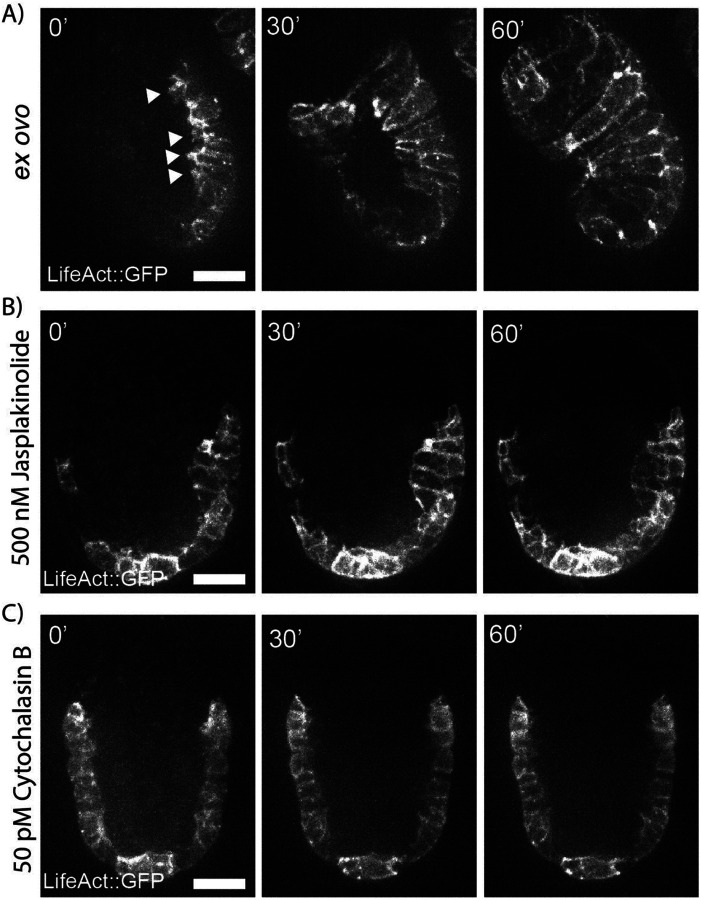
A) Maximum projection of *ex ovo* embryos in the absence of drug. B) Maximum projection of *ex ovo* embryos following treatment with 500 nm Jasplakinolide. C) Maximum projection of *ex ovo* embryos following treatment with 0.05 nM Cytochalasin B.

The actin cytoskeleton regulates many active processes in cell shape change and migration. The regulation of actin polymerization and branching generates mechanical anisotropies that direct a wide range of developmental events and has been extensively studied in the context of hypodermal enclosure in *C. elegans*^40,41^. Following gastrulation, hypodermal cells migrate towards the midline to enclose the embryo by extending protrusions along the leading edge (Fig 4A). Both the microtubule cytoskeleton and the assembly of contractile actin-myosin filaments are needed for proper enclosure. Embryos that fail to properly enclose typically rupture and arrest^41,42^.

Cytochalasin B is an inhibitor of actin polymerization by binding to F-actin and physically blocking filament extension. Laser ablation of the eggshell has previously been used to permeabilize the embryos to Cytochalasin B to determine actins' role in ventral enclosure and the migration of the leading cells^40^. In the absence of drug, *ex ovo* embryos conduct ventral enclosure normally (Fig 4A). The addition of 200 nM Cytochalasin B to embryos with an intact eggshell results in no visible phenotype. We conducted serial dilutions of the drug with *ex ovo* embryos and determined that even exceedingly low doses (50 pM) result in the failure of the leading cells to enclose the embryo (Fig 4B).

Jasplakinolide acts in the opposite manner of cytochalasin B by binding to and stabilizing f-actin. It has been used to implicate F-actin contractile rings in the differentiation of spermatid^43^. Starting with a dose half of that used in adult worms (500 nM), all embryos assayed ventral enclosure was completely halted with no migration of the leading cells or any of the other epidermal cells towards the midline (Fig 4C). Embryos with intact eggshells had no visible defects and enclosed normally. Low doses of the drug (10 nM and 1 nM) still block the migration of the leading cells, not allowing for the embryo to enclose and leading to lysis of the embryo.

### The impacts of dynein activity on the structure of centrosomes

Centrosomes must maintain structural integrity under high levels of mechanical stress during mitosis. Cytoplasmic dynein plays an important role in shuttling proteins from the cytoplasm to the pericentriolar material to maintain centrosome integrity and to mediate centrosome duplication^44,45^. Previous studies have shown that inhibiting cytoplasmic dynein will cause cells to fail to divide and centrosomes will lose structural integrity leading to an increase in the size of the pericentriolar material^44,46^.

**Figure 5.**
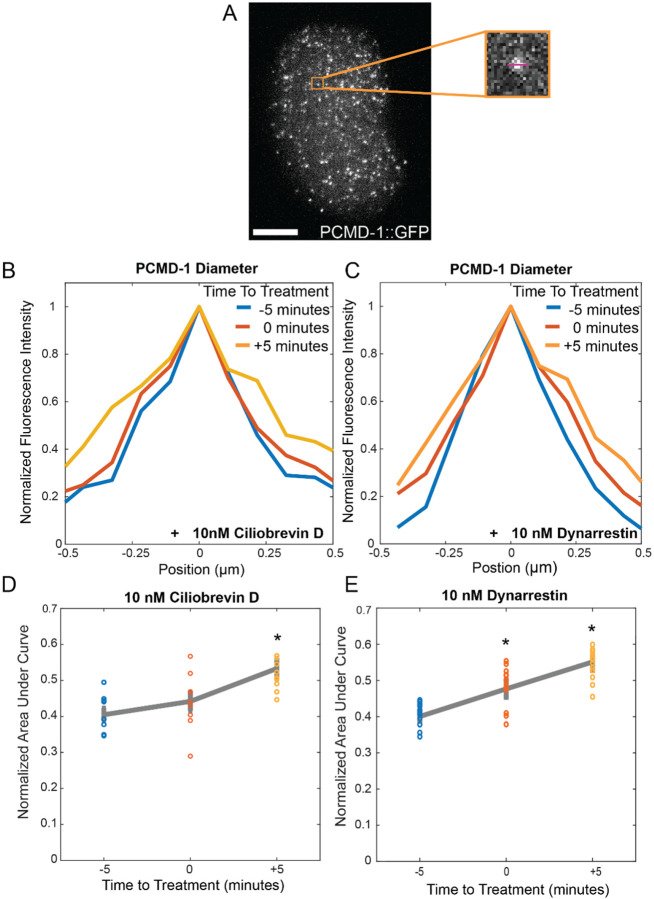
A) maximum projection of PCMD-1::GFP embryo. Inset showing one centrosome with a line through to demonstrate the line scan measurements taken. B) Line plot of PCMD-1 diameter for ciliobrevin D (left) and dynarrestin (right) at five minutes before drug addition (blue), at the time of drug addition (blue) and five minutes post drug addition (orange). C) Line plots of the normalized area under the curve for both ciliobrevin D (left) and dynarrestin (right) at five minutes before drug addition (blue), at the time of drug addition (blue) and five minutes post drug addition (orange). Stars denote significance.

Ciliobrevin D inhibits dynein’s ATPase activity, thus blocking its processivity^47^. We treated *ex ovo* embryos with 10 nM Ciliobrevin D and tracked centrosome size in interphase cells using an endogenously tagged allele of PCMD-1^48^. We measured centrosome size by integrating the area under a uniformly sized line profile through centrosomes 5 minutes prior to drug addition, immediately following drug addition, and 5 minutes post-drug addition, observing a significant increase in PCM area (p= 4.9 × 10^−4^) (Fig 5A–C).

Dynarrestin blocks the ability of dynein to bind to microtubules without affecting ATP hydrolysis^46^. We treated embryos with 10 nM Dynarrestin in the same manner as Ciliobrevin D and tracked centrosome size (Fig 5b). Treated embryos show a significant increase in centrosome size immediately (<1 minute) following drug addition (p= 0.003) as well as five minutes post drug addition (p= 1.7 × 10^−5^) (Fig 5C).

## Discussion

The study of cell biology has long made use of an assorted toolkit of highly specific toxins, inhibitors, and fluorescent dyes to manipulate and illuminate biology molecular processes *in vivo*. The impermeability of the *C. elegans* eggshell has long hampered the adoption of these tools for the study of developmental processes in a valuable model organism, motivating several efforts to overcome these limitations. The discovery of *perm-1*, and its inhibition by RNAi to render the early embryo permeable to small molecules was a revolutionary step forward in this field, and has been used to dissect diverse processes in early embryonic development. While this method is practical for early embryo studies, it is not practical for studies that look at events taking place after gastrulation due to the low brood size following RNAi and the low viability of embryos following gastrulation. Culturing *perm-1* RNAi embryos extended their viability, but their inability to develop past gastrulation prevents their use for the study of later developmental processes. Still, we saw that culturing *perm-1* RNAi embryos in L-15 + sucrose media extended their survival by several rounds of cell division, thus a combination of the two will likely be useful for studies of pre-gastrulation embryogenesis.

Using LysoTracker Red and BDP 630/650, we examined the distribution of lysosomes and lipids within the embryo over time. We observed an increase in the localization of both Lysotracker and BDP puncta in the gut region during the bean to comma transition period. This period coincides with the polarization of the gut lumen and transcriptional signatures of endodermal maturation, in particular the expression of a range of catabolic enzymes linked to gut metabolism such as the intestinal acid phosphatase *pho-1*^49^, raising the possibility that these changes in lysosome and neutral lipid levels in the embryonic gut may be linked to the initiation of increased intestinal metabolism.

Taxol is a potent stabilizer of microtubules, treatment of *ex ovo* embryos leads to a dramatic increase in cortical and spindle-associated tubulin at all embryonic stages. Colchicine, on the other hand, exhibits both stage and concentration-dependent variation in its effects. At a high dose (200 nM) it is a potent destabilizer of microtubules, causing steady loss of cortical localization and subsequent swelling of treated embryos. At an intermediate dose (10 nM) we observed stabilization of both cortical and spindle-associated microtubules in gastrulating embryos and destabilization in comma stage embryos while at a lower dose (1 nM) we observe stabilization, yet only of spindle-associated microtubules. Whether this reflects stage-dependent variation in the localization of tubulin isotypes or specific drug-mediated effects is unclear.

Mechanical permeabilization of embryos has previously been used to introduce actin-targeting toxins into *C. elegans* embryos to manipulate hypodermal enclosure^40,41^. Both depolymerization and stabilization of filamentous actin in the enclosing hypodermis results in rapid cessation of enclosure and embryonic arrest. Cytochalasin B, an inhibitor of actin polymerization, blocked the leading cells from extending cellular projections and ventral migration. Jasplakinolide, a stabilizer of actin filaments, also leads to the embryo failing to properly enclose, demonstrating the importance of actin dynamics in cell motility.

The pericentriolar material (PCM) is a highly structured proteinaceous matrix that is actively regulated in its compostion^50,51^. The microtubule minus-end-directed motor protein dynein plays a critical role in trafficking PCM components to the centrosome and maintaining the density of the PCM shell^45^. Inhibition of cytoplasmic dynein has previously been shown to result in an expansion of the PCM as this internally directed force is removed^44,46^. We show that, in *C. elegans*, inhibition of dynein through two distinct mechanisms, Ciliobrevin D which inhibits dynein’s ATPase activity and Dynarrestin which blocks microtubule binding, both lead to an increase in PCM size.

The generation of *ex ovo* embryos and their stable long-term culture will be a useful tool for cell and developmental biology, enabling the adoption of previously inaccessible tools in the study of *C. elegans* embryonic development. We have demonstrated that a wide range of dyes and drugs work effectively in these culture conditions at picomolar to nanomolar concentrations, often far lower than has been necessary via other permeabilization strategies. This approach is highly scalable, allowing the generation of large numbers of *ex ovo* embryos, and can be easily multiplexed with transgenics, mutants, and RNAi treatments since our approach is genotype-agnostic.

## Methods

### *C. elegans* strains

**Table:** 

Strain	Genotype	Source
FT1197	xnIs449 [lin-26::lifeAct::GFP + unc-119(+)]	CGC
N2		CGC
JH2054	axIs1492 [pie-1p::GFP::tbb-2 ORF::tbb-2 3'utr + unc-119(+)]	CGC
TMD119	pcmd-1(syb486[gfp::pcmd-1]) l	CGC
zyls36	zyls36 [cnd-1p::PH::mCherry; myo-2p::mCherry] X	Shah & Tanner *et al*. 2017^52^
PKS19	zyls36, unc-119(ed3) III; axIs1492.	This paper
PKS27	zyls36, pcmd-1(syb486[gfp::pcmd-1]) l	This paper

### Embryo collection

Supplemented media was prepared by dissolving 7.7 g sucrose into a 500 mL bottle of commercial L-15 media. The supplemented media was stored in 10 mL aliquots frozen at −20 °C. At this temperature, the media did not appear to degrade over time (for a period of at least 6 months). After thawing, if the media was used for long-term culture experiments (longer than approximately 2–3 hours), penicillin/streptomycin (100 units/mL final concentration) was added. Adding antibiotics prior to freezing resulted in precipitation that could interfere with imaging but did not appear to affect embryo viability.

Adult worms were seeded onto an NGM media plate for at least two days to obtain enough embryos. The plate containing embryos was washed off by pipetting 3000 μL M9 buffer and collecting it into a 15 mL centrifuge tube. The buffer was repeatedly drawn up and ejected onto the plate to completely collect worms. If residual worms and embryos remained on the plate post-washing, an additional 1000 μL of M9 was used. Enough M9 buffer was then added to the centrifuge tube containing worms and embryos to reach the 5 mL line. The contents were vortexed and centrifuged at 2,000 rpm (600 rcf) for 6 seconds to gently pellet the worms and embryos. The supernatant was aspirated down to the 1–2 mL line, taking care not to disturb the pellet. The pellet was washed again with 5 mL M9 solution three times as described above to remove bacteria.

5 mL of M9 buffer was added to the pellet. Then, 250 μL of 10–15% NaOCl (Millipore Sigma #425044) and 125 μL of 5N NaOH were added. The tube was vortexed in 30–60 second increments for 1–2 minutes. Embryos were pelleted by centrifuging at 2.0 rpm for 1 minute. The supernatant was aspirated, taking care not to disturb the pellet, and another round of the bleaching process was performed as described. After each vortexing step, the sample was checked under a dissection scope to confirm whether embryos were released from gravid adults and all worms fully digested to minimize debris carried over into subsequent steps. The bleached embryos were washed with 5 mL of the supplemented L-15 media three times to ensure complete removal of bleach.

### Eggshell digestion and embryo culture

After the final wash, embryos were pelleted again and excess media was removed. 50 μL of the embryos remaining were transferred to a microcentrifuge tube, followed by 10 μL of 10 mg/mL Yatalase (Takara T017). If processing fewer than 100–200 embryos, a single 10 μL Yatalase aliquot was added and incubated on a rocking rack for 1 hour before proceeding. If processing up to approximately 500 embryos, the suspension was incubated for 20 minutes while rocking after the first 10 μL Yatalase addition, and then left to stand for 10 minutes to allow the pellets to settle by gravity. Then, 10 μL of supernatant was carefully pipetted from the surface and examined under a microscope for the presence of embryos. If no embryos were observed, a total of 15 μL of the supernatant was removed . A second 10 μL Yatalase aliquot was then added to the tube and mixed by pipetting up and down. The sample was not vortexed at this stage, as partial digestion from the first Yatalase addition makes embryos fragile and prone to rupture. The embryos were incubated with the second Yatalase treatment for another 20 minutes on the rocking rack. After this stage, we do not wash the remaining Yatalase from the solution to minimize handling.

To verify the viability of *ex ovo* embryos, we transfer 10–20 embryos into 50 μL of fresh media in a 35 mm petri dish, cover the droplet with mineral oil to prevent evaporation, and leave the embryos at room temperature overnight, scoring survival based on the appearance of swimming L1 larvae the following morning.

### RNAi treatment

For *perm-1* RNAi by feeding, the clone targeting T01H3.4 from the Ahringer library was grown in 200ul of LB + carbenicillin overnight at 37 °C^53^. 100ul of bacterial culture was added to each of the 1 mM IPTG NGM plates and left to dry overnight. 20–40 L4 or young adult stage worms were added to each plate and left in a 20C incubator overnight.

For imaging, worms were transferred to a microwell slide in 50ul of 0.7x Egg Salts (1X Egg Salts: 118 mM NaCl, 40 mM KCl, 3.4 mM MgCl2, 3.4 mM CaCl2, 5 mM HEPES pH 7.4)^54^ or supplemented L-15 media. 5 worms were cut using a scalpel and an eyelash was used to separate out 2–4 cell embryos. Those embryos were transferred to 24x 50 mm coverslips and mounted using ~5ul of either 0.7x Egg salts or supplemented L-15 media with 20um beads. A top coverslip was placed and the edges sealed with molten petroleum jelly. Embryos were imaged for 4 hours to assess viability and progression through embryonic development.

### Light microscopy

*Ex ovo* embryos are extremely fragile mechanically and care should be taken to minimize direct manipulation, but are compatible with standard nematode embryo mounting practices such as bead-based compression mounting. Compression mounting with agar pads invariably results in the destruction of the embryos and should be avoided. We evaluated a handful of alternate sample mounting strategies in cases where the timing of drug introduction is desirable for temporal control of treatments for inverted high resolution imaging.

All imaging was performed using an Olympus IX83 inverted microscope frame, a UPSAPO 60XS2 silicone oil immersion objective and a VisiTech iSIM super resolution multipoint scanning confocal microscope.

### Image processing

Unless otherwise noted, no background subtraction or post-processing beyond linear contrast and brightness adjustment in Fiji was performed. Lysotracker imaging at low concentrations was prone to photobleaching so an exponential fit using Fiji’s bleach correction plugin was used to process these images.

### Bead mounting for imaging with inverted microscopes

*Ex ovo* embryos tolerate compressed mounting using methods previously optimized for imaging during long-term time lapse for cell lineage tracing^55^. Briefly, a suspension of 20 μm diameter polystyrene beads (Thermo Scientific 09–980-133) is prepared in L-15 media with sucrose and antibiotics. This preparation is diluted empirically to achieve a final concentration of 100–200 beads per μL. For imaging, *ex ovo* embryos are transferred into a 2 μL drop of bead suspension on a #1.5 coverslip by capillary micropipette. A smaller coverslip is placed on top and secured in place by painting with melted petroleum jelly without moving the coverslip after placement to avoid damaging the embryos. Imaging post-twitching embryos in this format is made challenging as the lack of a rigid eggshell allows *ex ovo* embryos to generate traction against the coverslip surfaces and migrate slowly.

### Open mounting for accessibility for drug delivery

While bead-compressed mounting is advantageous for numerous applications, the closed nature of the preparation complicates acute addition of small molecules. For the timed treatment of embryos with the dynein inhibitors Dynarrestin and Ciliobrevin B, we used open-chambered slides (ibidi 80821) to mount embryos. Mounting embryos in 50 μL of media and diluting additives to 100✕ stock concentrations to reduce the volume of the added drug minimized disruption to the embryo positions during time lapse imaging, allowing for precisely timed drug addition.
